# Two-Dimensional Numerical Study of Methane-Air Combustion Within Catalytic and Non-catalytic Porous Medium

**DOI:** 10.3389/fchem.2020.511792

**Published:** 2020-11-06

**Authors:** H. B. Gao, S. C. Zong, X. B. Feng, C. W. Zhang

**Affiliations:** ^1^College of Mechanical Engineering, Xi'an University of Science and Technology, Xi'an, China; ^2^Institute for Health and Environment, Chongqing University of Science and Technology, Chongqing, China; ^3^Shaanxi Key Laboratory of Safety and Durability of Concrete, Xijing University, Xi'an, China

**Keywords:** premixed combustion, catalytic, inert, porous media, packed bed

## Abstract

This study numerically investigates a two-dimensional physical model of methane/air mixture combustion in catalytic and non-catalytic porous media. The temperature distribution and flame stability of combustion in inert alumina (Al_2_O_3_) pellets and platinum (Pt) catalyst-supported alumina (Al_2_O_3_) pellets, were studied by changing the burner structure, operating parameters, and physical properties of alumina pellets. The simulation results indicated that the gas temperature in the inert porous medium is higher than that in a catalytic porous medium, while the solid temperature in an inert porous medium is lower than that in a catalytic porous medium. The flame moved toward the burner exit with the increasing diameter of the packed pellets at a lower equivalence ratio and moved toward upstream with the increased thermal conductivity of packed pellets. The flame location of the catalytic porous burner was more sensitive to the flame velocity and insensitive to thermal conductivity compared to the inert porous burner. The distance of the flame location to the burner inlet is almost constant with the increasing length of the porous media for both the catalytic and inert porous burner, while the relative position of the flame location moved toward the upstream.

## Introduction

Low calorific value gases mainly include blast furnace gas, coke oven gas, coal seam gas, landfill gas, biogas, and other combustible gases (Wood and Harris, 2008; Mujeebu et al., 2009), which are regarded as “exhaust gas” and directly discharge to the environment. It is difficult to handle by conventional free flame combustion and can lead to environmental pollution and energy waste. The low calorific value gases are usually burned by preheating the mixture of air/fuel with an auxiliary device as assistant equipment. Porous media combustion is a promising method for handling low calorific value gases, which can recuperate heat from the combustion zone to the fresh mixture of fuel/air by solid conduction and radiation. It has been the subject of much attention in the past few decades due to its characteristics of higher power density, flame speeds, efficiency, and its lower pollutant emissions, compared with free flame combustion.

Much research has been done on the structures and materials of porous media to examine how they maintain good flame stabilization. Foe example, two layer burners with different porosity ceramic blocks were designed to extend the flame stability limits and reduce pollution, and Ellzey et al. (Mathis and Ellzey, 2003; Smucker and Ellzey, 2004; Vogel and Ellzey, 2005) found that the flame can easily stabilize at the interface of these different blocks. Hayashi et al. (2010) numerically studied the performance of a two-section burner with a perforated alumina (Al_2_O_3_) plate and 10 ppi SiC porous foam and indicated that the flashback can be avoided by using porous media with smaller pore diameter. Gao et al. (2011, 2012, 2014) experimentally studied the performance of two-layer porous burners filled with different diameter alumina (Al_2_O_3_) beads and foams of different materials. This indicated that the flame stability limit and the maximum flame temperature could be increased with the increasing diameter of packed beads, while the foam materials have little effect on the monoxide (CO) emission. Furthermore, Gao et al. (2017) experimentally studied the effect of the gap between alumina (Al_2_O_3_) pellets (preheating zone) and silicon carbide (SiC) foam (combustion zone) on the performance of methane/air combustion and reported that the limits of flame stability could be extended with a proper gap length. Hashemi and Hashemi (2017) reported that flame stability limits and flame temperature in a two-layer porous burner can be controlled by the equivalent ratio of methane/air mixture. Wang et al. (2018) studied the fuel-rich combustion of methane (CH_4_) in a double-layer porous burner filled with alumina (Al_2_O_3_) beads of different diameters. An optimal pellet diameter of 7.5 mm at the downstream was obtained with the highest syngas energy conversion efficiency. Liu et al. (2019) investigated the extra-lean filtration combustion of propane/air in porous media and showed that the average flame velocity increased and the temperature difference between the solid and gas becomes smaller.

Catalytic combustion of fuel/air mixture within a porous media reactor is an approach that can enhance combustion efficiency and decrease pollutant emissions, as the porous media acts as a special reaction place and supports the catalyst. There are few studies focused on combustion in catalytic and non-catalytic porous media. Most of the previous research is focused on combustion in catalytic monolith type burners with different channel geometries (Groppi and Tronconi, 1997; Cybulski and Moulijn, 1998; Goralski and Schmidt, 1999). Rumminger et al. (1999) attempted to increase the operating range and stability of the burner by employing platinum and/or palladium to the porous media. Wierzba and co-workers (Younis and Wierzba, 2004; Shahamiri and Wierzba, 2009) developed the model of combustion of methane within both the catalytic and non-catalytic packed-bed reactors by employing single-step and multi- step reaction mechanisms for both gas-phase (homogeneous) and catalytic surface (heterogeneous) reactions. Shahamiri and Wierzba (2010) further studied the effect of the addition of hydrogen to the biogas/air mixture on combustion in a catalytic porous burner using detailed surface chemistry, which indicated that hydrogen can improve the oxidation of methane. Zhong et al. (2015) experimentally studied the effect of catalyst length on the combustion characteristics of n-butane and showed that the lower flame stability limits did not extend with various lengths of the catalyst. Yang et al. (2015) studied the combustion of methane (CH_4_) in nickel (Ni) foam supported Pd/Al_2_O_3_ catalyst and proved that the nickel (Ni) foam supported the Pd/Al_2_O_3_ catalyst, with better performance. Qu and Feng (2015), Feng and Qu (2016) studied the catalytic combustion of premixed methane/air in a two-zone perovskite-based alumina (Al_2_O_3_) pileup-pellets burner, and indicated that the flame stability limits increased with the increase of equivalence ratio or pellet diameter. Li et al. (2018) used a metal foam catalyst to catalyze methane (CH_4_) combustion in a micro-combustion chamber and investigated the effects of inlet velocity and equivalence ratio on catalytic combustion characteristics of methane. These results show that a mixture of methane/air has an equivalent ratio of 1.0 and that a mixed fuel with an inlet velocity of 0.2–0.6 m/s can achieve combustion. Yang et al. (2020) numerically simulated the uniform combustion characteristics of methane/air in a semi-packed bed catalytic combustion chamber and found three combustion modes: completely heterogeneous combustion, heterogeneous combustion, and incomplete heterogeneous combustion.

Many models have been proposed for simulating combustion in catalytic porous media. However, most previous works were one-dimensional models for the combustion of methane/air mixtures in catalytic porous media burner. The main objective of the present work is to develop a two-dimensional model for the combustion of methane/air mixtures in a catalytic porous media burner by considering both gas-phase (homogeneous) and catalytic surface (heterogeneous) reactions, which were compared with combustion in an inert porous burner, and modeled as single-step global reactions of Arrhenius type.

## Physical Model and Governing Equations

**Figure 1:**
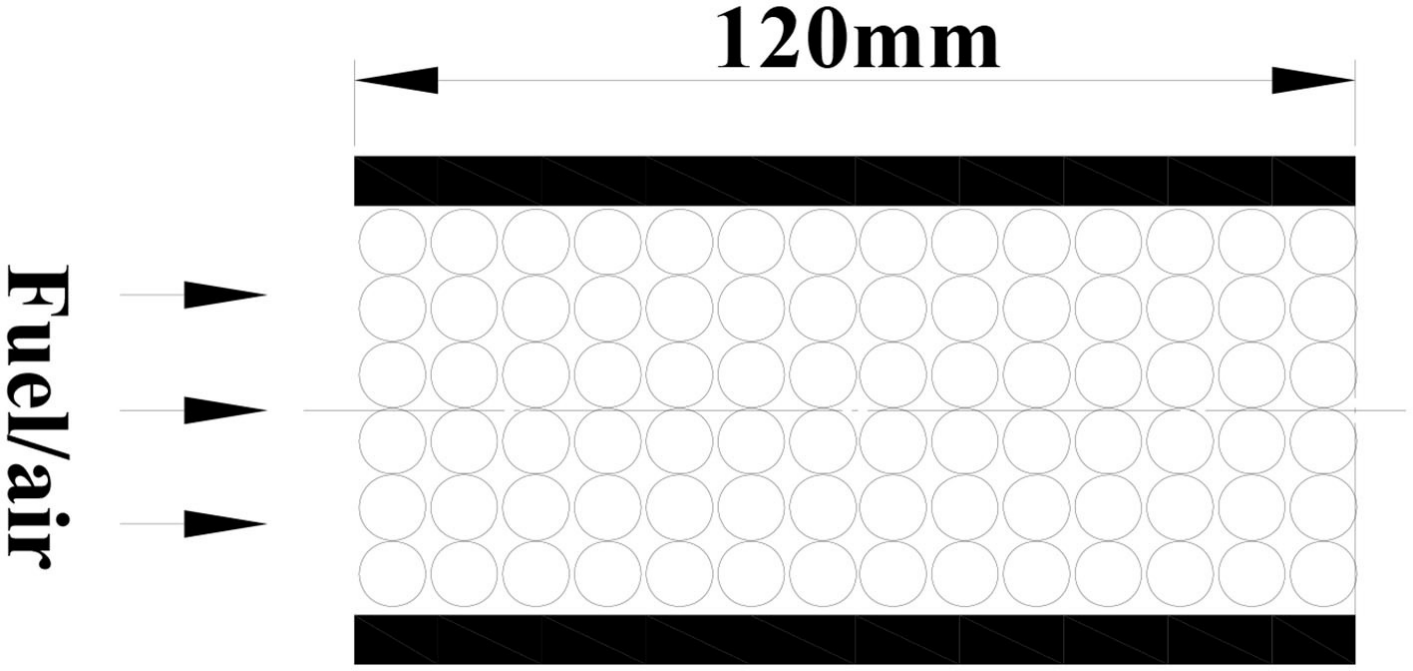
Schematic diagram of the physical model.

The porous burner is a cylinder with a length of 120 mm and a diameter of 50 mm, as shown in Figure 1, which are filled with different diameter inert alumina pellets or alumina pellets supported with Pt catalyst. In the present model, the fuel/air mixture and products are regarded as incompressible ideal gases. The gas radiation and Dufour effect in porous media are ignored. The thermophysical properties of the gases, such as the density, specific heat, and thermal conductivity, are considered to be variable and are evaluated for the instantaneous local temperature and mixture composition. The thermal properties of the pellets, such as the density, specific heat, and porosity, are assumed to be uniform and constant. The gas and solid assumed thermal non-equilibrium and two energy equations were employed. Inert porous combustion only contains gas phase reaction (homogeneous reaction), while catalytic porous combustion includes gas phase reaction (homogeneous reaction) and catalytic surface reactions (heterogeneous reaction). Both gas phase and catalytic surface reactions are modeled as single-step global reactions of Arrhenius type. The kinetic model of catalytic surface reaction (heterogeneous reaction) is based on the single step irreversible reaction of methane on a platinum surface by Song et al. (1991) and Markatou et al. (1993). The two-dimensional physical model and corresponding equations are as follows:

Continuity equation

(1) $$\begin{array}{l} \frac{\partial \left(\varepsilon \rho_{g}\right)}{\partial \tau }+\frac{\partial \left(\varepsilon \rho_{g}u_{g}\right)}{\partial x}+\frac{1}{r}\frac{\partial \left(\varepsilon \rho_{g}rv_{g}\right)}{\partial r}=0 \end{array}$$

Axial momentum equation

(2) $$\begin{array}{l} \frac{\partial \left(\varepsilon \rho_{g}u_{g}\right)}{\partial \tau }+\frac{\partial \left(\varepsilon \rho_{g}u_{g}u_{g}\right)}{\partial x}+\frac{1}{r}\frac{\partial \left(\varepsilon \rho_{g}ru_{g}v_{g}\right)}{\partial r}\\ =-\varepsilon \frac{\partial p}{\partial x}+\frac{\partial }{\partial x}\left(\mu \frac{\partial u_{g}}{\partial x}\right)+\frac{1}{r}\left(\mu r\frac{\partial u_{g}}{\partial r}\right)-R_{x} \end{array}$$

Radial momentum equation

(3) $$\begin{array}{l} \frac{\partial \left(\varepsilon \rho_{g}v_{g}\right)}{\partial \tau }+\frac{\partial \left(\varepsilon \rho_{g}u_{g}v_{g}\right)}{\partial x}+\frac{1}{r}\frac{\partial \left(\varepsilon \rho_{g}rv_{g}v_{g}\right)}{\partial r}\\ =-\varepsilon \frac{\partial p}{\partial x}+\frac{\partial }{\partial x}\left(\mu \frac{\partial v_{g}}{\partial x}\right)+\frac{1}{r}\left(\mu r\frac{\partial v_{g}}{\partial r}\right)-R_{r} \end{array}$$

Gas energy equation

(4) $$\begin{array}{l} \frac{\partial \left(\varepsilon \rho_{g}c_{g}T_{g}\right)}{\partial \tau }+\frac{\partial \left(\varepsilon \rho_{g}c_{g}u_{g}T_{g}\right)}{\partial x}+\frac{1}{r}\frac{\partial \left(\varepsilon \rho_{g}c_{g}rv_{g}T_{g}\right)}{\partial r}\\ +\overset{N}{\underset{k=1}{\sum }}\varepsilon \rho_{g}c_{g}Y_{k}V_{k}\frac{\partial T_{g}}{\partial x}+\overset{N}{\underset{k=1}{\sum }}\varepsilon \rho_{g}c_{g}Y_{k}V_{k}\frac{1}{r}\frac{\partial \left(rT_{g}\right)}{\partial r}\\ =\frac{\partial }{\partial x}\left(\varepsilon k_{g}\frac{\partial T_{g}}{\partial x}\right)+\frac{\partial }{\partial r}\left(\varepsilon k_{g}\frac{\partial \left(rT_{g}\right)}{\partial r}\right)\\ +\left(1-\varepsilon \right)h_{v}\left(T_{s}-T_{g}\right)-\varepsilon \overset{N}{\underset{k=1}{\sum }}{\overset{∙}{\omega }}_{k}H_{k}W_{k} \end{array}$$

Inert solid energy equation

(5) $$\begin{array}{l} \frac{\partial }{\partial \tau }\left[\left(1-\varepsilon \right)\rho_{s}c_{s}T_{s}\right]=\frac{\partial }{\partial x}\left[\left(1-\varepsilon \right)\left(k_{s}+k_{rad}\right)\frac{\partial T_{s}}{\partial x}\right]\\ +\frac{1}{r}\frac{\partial }{\partial r}\left[\left(1-\varepsilon \right)\left(k_{s}+k_{rad}\right)\frac{\partial T_{s}}{\partial r}\right]+\left(1-\varepsilon \right)h_{v}\left(T_{g}-T_{s}\right) \end{array}$$

Catalytic solid energy equation

(6) $$\begin{array}{l} \frac{\partial }{\partial \tau }\left[\left(1-\varepsilon \right)\rho_{s}c_{s}T_{s}\right]=\frac{\partial }{\partial x}\left[\left(1-\varepsilon \right)\left(k_{s}+k_{rad}\right)\frac{\partial T_{s}}{\partial x}\right]\\ +\frac{1}{r}\frac{\partial }{\partial r}\left[\left(1-\varepsilon \right)\left(k_{s}+k_{rad}\right)\frac{\partial T_{s}}{\partial r}\right]\\ +\left(1-\varepsilon \right)h_{v}\left(T_{g}-T_{s}\right)+\left(1-\varepsilon \right){\overset{∙}{\omega }}_{k}^{s}H_{k}W_{k}a_{cat} \end{array}$$

Component equation

(7) $$\begin{array}{l} \frac{\partial }{\partial \tau }\left(\varepsilon \rho_{g}Y_{k}\right)+\frac{\partial }{\partial x}\left(\varepsilon \rho_{g}Y_{k}u_{g}\right)+\frac{1}{r}\frac{\partial }{\partial r}\left(\varepsilon \rho_{g}Y_{k}rv_{g}\right)\\ =-\frac{\partial }{\partial x}\left(\varepsilon \rho_{g}Y_{k}V_{k}\right)-\frac{1}{r}\frac{\partial }{\partial r}\left(\varepsilon \rho_{g}Y_{k}rV_{k}\right)+\varepsilon {\overset{∙}{\omega }}_{k}W_{k} \end{array}$$

Gas state equation

(8) $$\begin{array}{l} \rho_{g}=\frac{pW_{m}}{R_{u}T_{g}}\;W_{m}=\frac{1}{\overset{N}{\underset{k=1}{\sum }}\frac{Y_{k}}{W_{k}}} \end{array}$$

Homogenerous reaction equation

(9) $$\begin{array}{l} \omega^{h}=A_{g}C_{CH_{4}}^{0.2}C_{O_{2}}^{1.3}\exp\left(-\frac{E_{a}}{R_{u}T_{g}}\right) \end{array}$$

Hetergenerous reaction equation

(10) $$\begin{array}{l} \omega^{s}=A_{s}C_{CH_{4}}C_{O_{2}}^{0.5}\exp\left(-\frac{E_{s}}{R_{u}T_{s}}\right) \end{array}$$

The relationship of molar concentration *C*_*k*_ and mass fraction *Y*_*k*_ of the species is calculated from

(11) $$\begin{array}{l} C_{k}=\rho_{g}\frac{Y_{k}}{W_{k}} \end{array}$$

Where *T*_*s*_, *K*_*s*_, *C*_*s*_, and ρ_*s*_ are temperature, thermal conductivity, specific heat, and density of packed pellet, *T*_*g*_, *k*_*g*_, *c*_*g*_, and ρ_*g*_ are temperature, thermal conductivity, specific heat and density of the gas mixture, *k*_*rad*_ is equivalent to the radiation heat transfer coefficient of the thermal conductivity of the gas mixture and the radiation of solid skeleton. *E*_*a*_ and *E*_*s*_ are the activation energy of the gas phase reaction (homogeneous reaction) and catalytic surface reactions (heterogeneous reaction), *A*_*g*_ and *A*_*s*_ are a pre-exponential factor of gas phase reaction and catalytic surface reactions, *V*_*k*_, *Y*_*k*_, *C*_*k*_, ${\overset{∙}{\omega }}_{k}$, *W*_*k*_, *H*_*k*_ is the diffusion rate, mass fraction, molar concentration, chemical reaction rate, molecular weight and enthalpy of formation of the k th species, respectively. *h*_*v*_ is the volumetric heat transfer coefficient between gas and porous skeleton. Table 1 shows the formulas for resistance term in the momentum equation, volumetric convective heat transfer coefficient between gas and solid in the gas energy equation, effective thermal conductivity in the solid energy equation, and the specific surface area of catalytic porous media. Gas-phase (homogeneous) and catalytic surface (heterogeneous) reactions are considered in the present work and the relevant parameters are shown in Table 2.

**Table 1:** Numerical calculation of physical parameters.

**Equation**	**Calculated** **amount**	**Calculation** **formula**	**References**
Axial momentum equation	*R*_*x*_	$R_{x}=\text{180}\frac{{\left(\text{1}-\varepsilon \right)}^{\text{2}}}{\varepsilon^{\text{3}}}\frac{\mu u_{g}}{d_{p}^{2}}+\text{1}\text{.8}\frac{\text{1}-\varepsilon }{\varepsilon^{\text{3}}}\frac{\rho |v|u_{g}}{d_{p}}$	Yang et al., 2020
Radial momentum equation	*R*_*r*_	$R_{r}=\text{180}\frac{{\left(\text{1}-\varepsilon \right)}^{\text{2}}}{\varepsilon^{\text{3}}}\frac{\mu v_{g}}{d_{p}^{2}}+\text{1}\text{.8}\frac{\text{1}-\varepsilon }{\varepsilon^{\text{3}}}\frac{\rho |v|v_{g}}{d_{p}}$	Wakao and Kaguei, 1982
Gas energy equation	*h*_*v*_	$h_{v}=\left(\frac{6\varepsilon }{d_{p}^{2}}\right)k_{g}Nu$ Nu=2.0+1.1Pr1/3Rep0.6 Pr=μcpkg,Rep=ρgεugdpμ	Modest, 1993
	*V*_*k*_	*V*_*k*_ = −*D*_*km*_∇*Y*_*k*_ $D_{km}=\frac{1-Y_{k}}{\sum_{j\neq k}^{N}X_{j}/D_{kj}}$	Kuo, 1986
Solid energy equation	*k_*rad*_*	$k_{rad}=\frac{32\varepsilon d_{p}\sigma T_{s}^{3}}{9\left(1-\varepsilon \right)}$	Dobrego et al., 2008b
Specific surface area	*a*_*cat*_	*a*_*cat*_ = 6(1−ε)/*d*_*p*_	Dobrego et al., 2008a

**Table 2:** Parameters in the present work.

**Parameter**	**Numerical value**
Fuel	CH_4_
Equivalent ratio	0.20–0.30
Speed	0.02–0.35 m·s^−1^
Burner diameter	0.05–0.10 m
Burner length	0.06–0.18 m
Bed type	0.5% Pt/γ-Al_2_O_3_
Thermal conductivity of pellets	0.2 W·m^−1^·K^−1^
Porosity	0.45–0.52

## Initial and Boundary Conditions

At the inlet

(12) $$\begin{array}{l} u=u_{in},v=0,T_{g}=T_{g,in},Y_{fu}=Y_{fu,in},Y_{ox}=Y_{ox,in},\frac{\partial T_{s}}{\partial x}=0 \end{array}$$

At the outlet

(13) $$\begin{array}{l} \frac{\partial u}{\partial x}=\frac{\partial v}{\partial x}=\frac{\partial T_{g}}{\partial x}=\frac{\partial Y_{fu}}{\partial x}=\frac{\partial Y_{ox}}{\partial x}=0 \end{array}$$

At the outlet for solid skeleton

(14) $$\begin{array}{l} \frac{\partial T_{s}}{\partial x}=0\;\left(\text{without radiative}\right) \end{array}$$

(15) $$\begin{array}{l} \left(1-\varepsilon \right)k_{s}\frac{\partial T_{s}}{\partial x}=-\varepsilon_{r}\sigma \left(T_{s}^{4}-T_{sur}^{4}\right)\left(\text{with radiative}\right) \end{array}$$

At the axis, symmetry condition

(16) $$\begin{array}{l} \frac{\partial u}{\partial r}=v=\frac{\partial T_{s}}{\partial r}=\frac{\partial T_{g}}{\partial r}=\frac{\partial Y_{fu}}{\partial r}=\frac{\partial Y_{ox}}{\partial r}=0 \end{array}$$

At the wall, no-slip, impenetrability, and adiabatic

(17) $$\begin{array}{l} \frac{\partial u}{\partial r}=\frac{\partial v}{\partial r}=\frac{\partial T_{s}}{\partial r}=\frac{\partial T_{g}}{\partial r}=\frac{\partial Y_{fu}}{\partial r}=\frac{\partial Y_{ox}}{\partial r}=0 \end{array}$$

In addition to the radiation at the burner exit, the wall boundary condition is one of the factors that affect the performance of the burner. In the present work, the wall boundary condition is equivalent to the thin-wall with constant external convective heat transfer coefficient

(18) $$\begin{array}{l} q_{loss}=h_{wall}\left(T_{wall}-T_{sur}\right) \end{array}$$

Where, *T*_*wall*_ and *T*_*sur*_ are the wall temperature and ambient temperature (300 K), respectively. The heat loss is equal to zero when the convective heat transfer coefficient *h*_*wall*_ → 0, which means adiabatic condition at the wall. The constant temperature condition at the wall was considered when the wall temperature *T*_*wall*_ is equal to the ambient temperature *T*_*sur*_.

## Numerical Methods

### Solution Procedure

The governing equations are solved by the commercial software Fluent 6.3 and a user defined scalar (UDS) was added to modify the original single temperature model into a two-temperature model. Moreover, user-defined function (UDF) was used to consider the catalytic surface (heterogeneous) reaction in the solid energy equation of catalytic combustion, volumetric heat transfer coefficient between gas and porous skeleton, the effective thermal conductivity of the solid skeleton by considering radiation. The pressure-velocity coupled equation was solved using the SIMPLEC (Semi-Implicit Method for Pressure-Linked Equations Couple) algorithm. The first-order upwind scheme and central difference scheme adopted for the convection term, and the diffusion term in the above differential equations. The under relaxation iteration was adopted due to the strong non-linearity of the chemical reaction. A temperature patch of 1,100 K was used to initiate the reaction. A smaller time step was adopted at the beginning of the iteration and the time step appropriately increased when the flow and temperature changes are negligible, the relevant time step was shown in Table 3. The residuals of conservation equations were monitored and the calculation was judged as convergence when the residual is <10^−6^.

**Table 3:** Time step of unsteady calculation.

**Time (s)**	**Time step, Δτ (s)**
τ <0.001	Δτ = 0.00001
0.001 < τ <0.01	Δτ = 0.0001
0.01 < τ <0.1	Δτ = 0.001
τ > 0.1	Δτ = 0.1

### Model Validation

**Figure 2:**
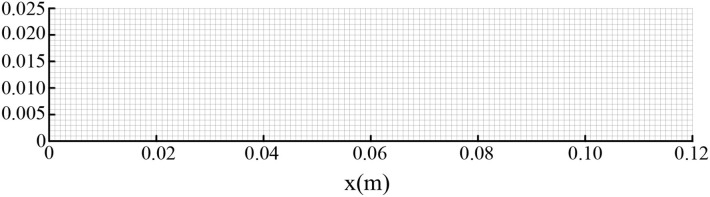
Schematic diagram of grid division.

**Figure 3:**
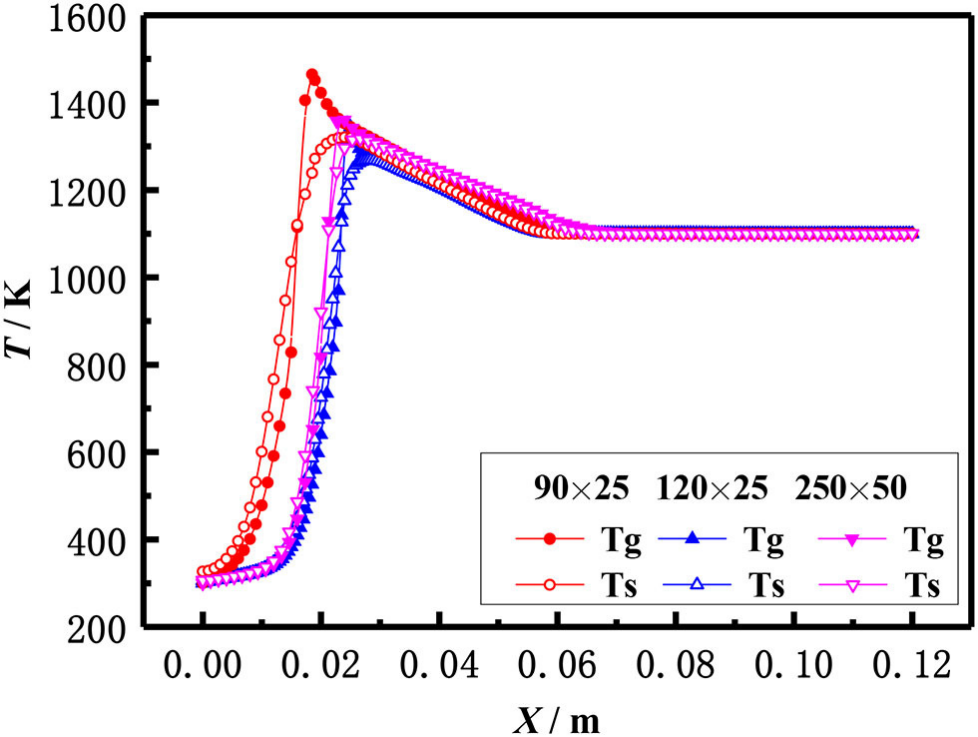
Grid independence check.

**Figure 4:**
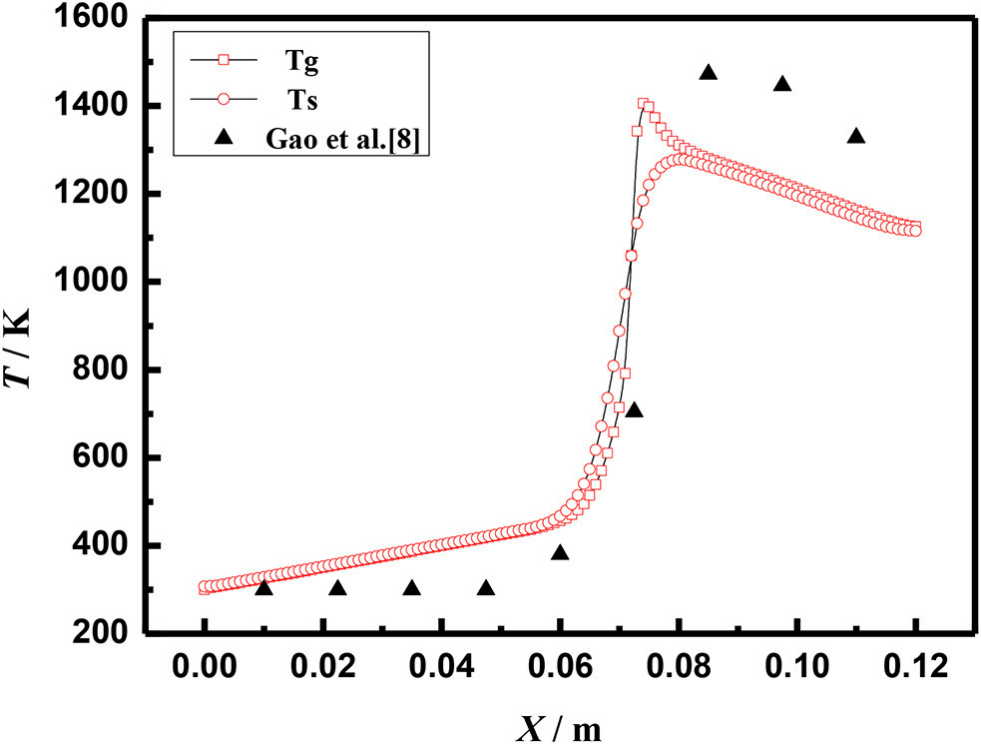
Comparison of temperature profiles (φ = 0.60, S = 30 cm/s).

The above equations are solved by the finite volume method (FVM), the burner is a symmetrical structure and the grid generation is shown in Figure 2. The grid independence of porous media burners with a 6 mm diameter packed bed were checked, as shown in Figure 3. The numerical results of different grid numbers are examined for combustion in inert porous media at the equivalence ratio of φ = 0.60 and the flow velocity of S = 30 cm/s, the grid of 120 × 25 is finally selected. Figure 4 shows the comparison of the temperature profiles between the numerical results and the experimental results of our previous work (Gao et al., 2012) with an equivalence ratio of 0.60 and flow velocity of 30 cm /s. The simulation results are in good agreement with the experimental results of Gao et al. (2012), which indicated that the numerical simulation model is reasonable.

**Figure 5:**
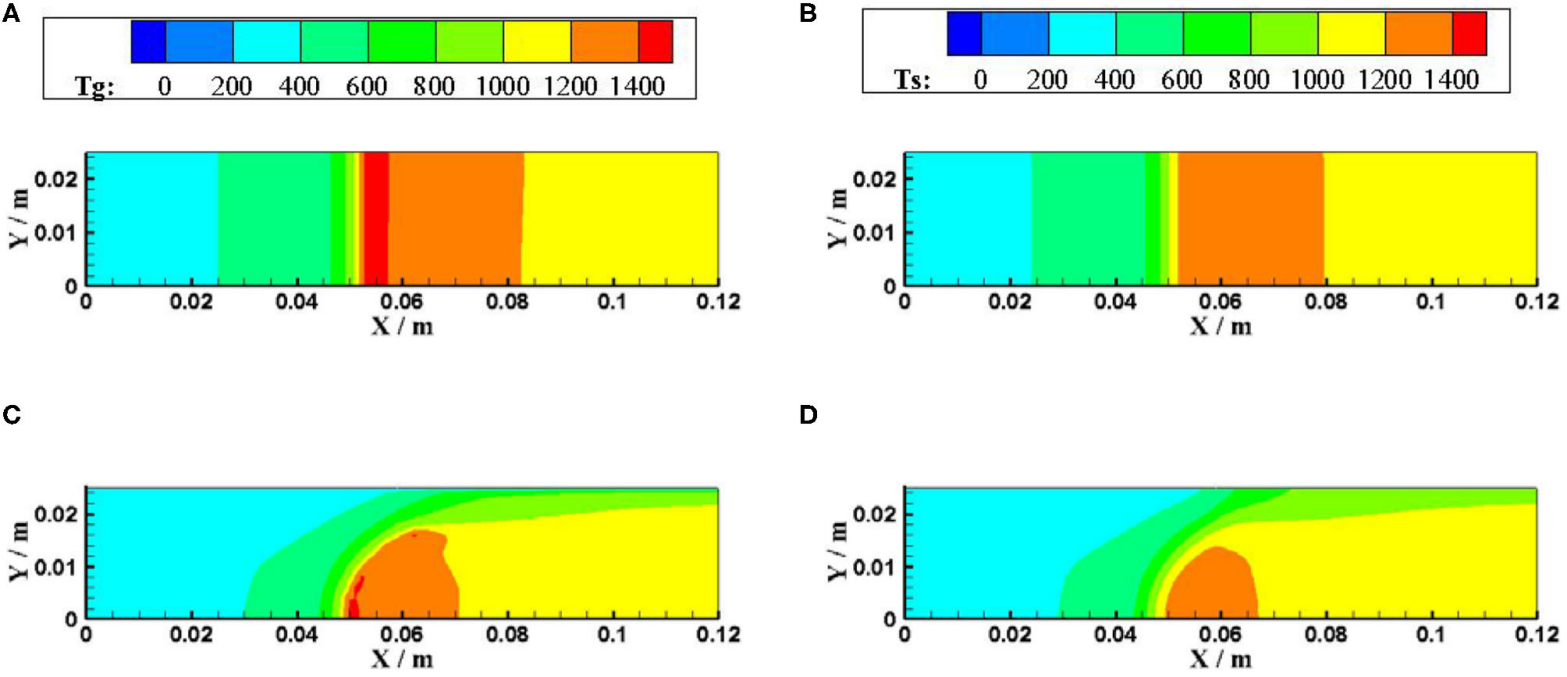
Temperature distributions for different boundary condition of the wall (φ = 0.60, S = 30 cm/s). **(A)** gas temperature for adiabatic. **(B)** solid temperature for adiabatic. **(C)** gas temperature for constant temperature. **(D)** solid temperature for constant temperature.

**Figure 6:**
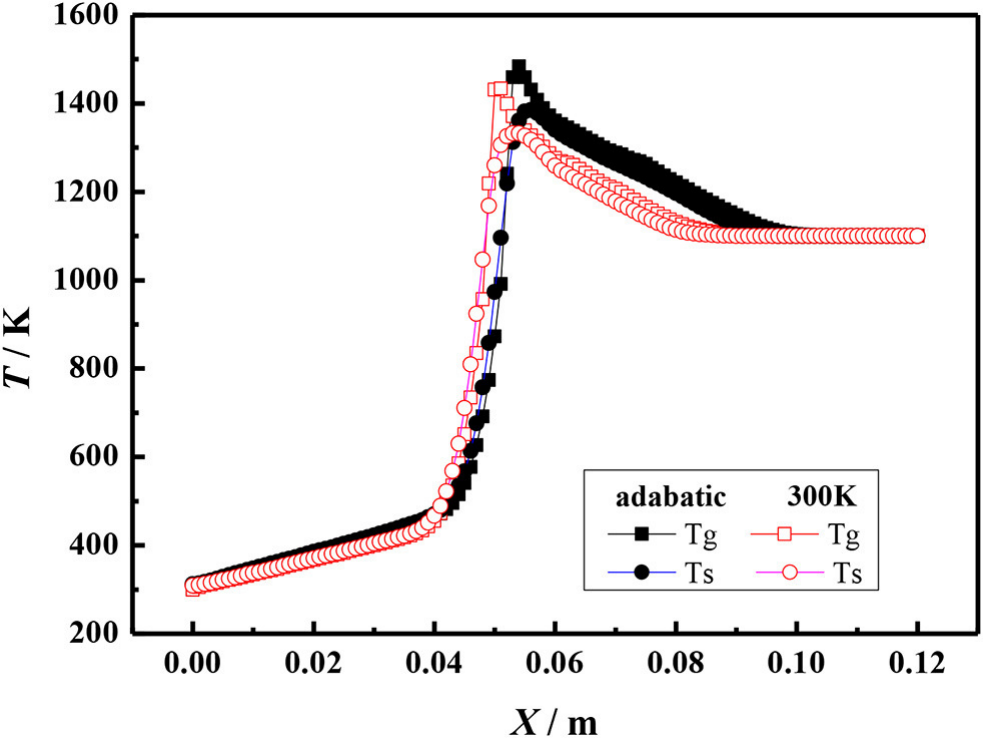
Temperature profiles for different boundary condition of the wall (φ = 0.60, S = 30 cm/s).

Figures 5, 6 show the gas and solid temperature distributions of a 3–6 mm packed bed double-layer porous burner at the equivalence ratio of φ = 0.60 and the flow velocity of S = 30 cm/s. It can be seen that the temperature distribution of gas and solid presents a two-dimensional structure, and the one-dimensional model cannot accurately describe the temperature fields of combustion. The temperature near the wall is the same as the axial centerline temperature when the wall is adiabatic. The wall temperature is lower than the axial centerline temperature when the wall has heat dissipation loss. The greater the heat loss of the wall, the greater the temperature difference between the wall and the axial centerline. Generally, the combustion region is shrunk with the increase of heat loss from the wall, and the flame moves to the upstream.

**Figure 7:**
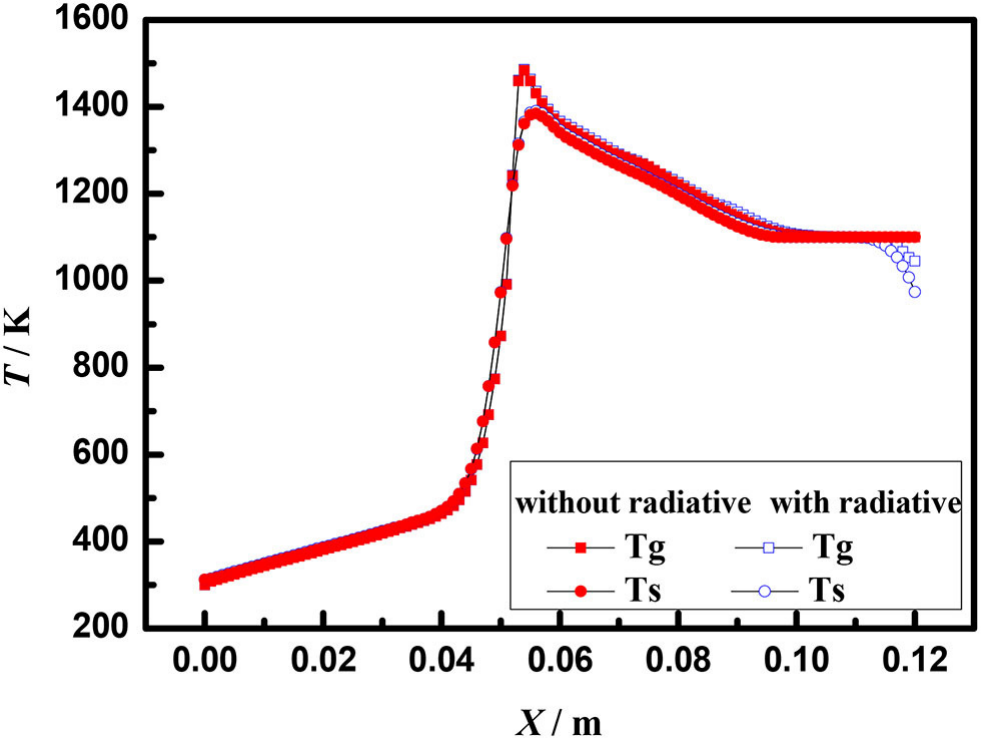
Temperature profiles of a 3–6 mm packed bed porous burner (φ = 0.60, S = 30 cm /s).

Figure 7 shows the axial temperature distribution of porous media burners with and without radiation at the burner exit at the equivalence ratio of φ = 0.60 and the flow velocity of S = 30 cm/s. Compared with the boundary condition without radiation, the flame location moves to the burner exit when the radiation boundary condition is considered at the burner exit. Moreover, the temperature of gas and solid at burner outlet with radiation is significantly lower than that without radiation. This indicates that the radiation influences the gas and solid temperature near the exit of the burner, but has little effect on the flame structure.

## Results and Discussions

### Flame Profile of Catalytic and Inert Porous Media

**Figure 8:**
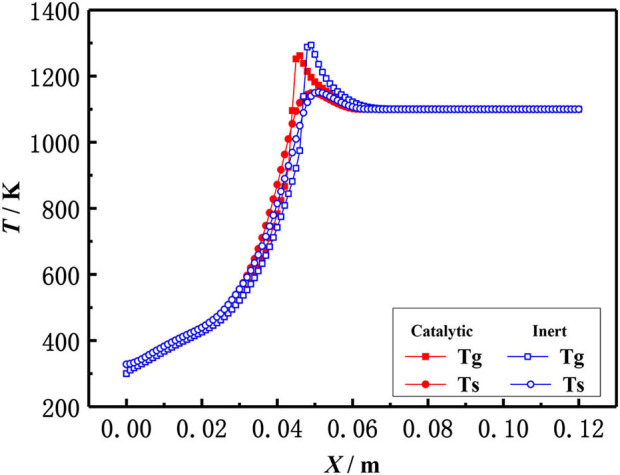
Temperature distribution of gas and solid in catalytic and inert porous media (φ = 0.30, S=20 cm/ s).

The effect of catalytic porous media and inert porous media on the flame temperature profile was studied in the present work. Figure 8 shows the flame temperature distributions of gas and solid in a 6 mm inert alumina (Al_2_O_3_) pellets and platinum (Pt) catalyst-supported alumina (Al_2_O_3_) pellets with an equivalence ratio of φ = 0.30 and an inlet velocity of S = 20 cm/s. In the combustion zone, the flue gas temperature is higher than that of a solid skeleton, the heat released from enthalpy release of gas combustion, and transferred to the solid skeleton by convection. The heat is then transferred to the upstream (preheating zone) by heat conduction and radiation through the solid skeleton, where the premixed fresh gas mixture is preheated in the solid skeleton by convection. The gas temperature in the inert porous medium was higher than that in the catalytic porous medium, while the solid temperature in the inert porous medium was lower than that in the catalytic porous medium. The profiles near the inlet and outlet are very similar because of the velocity, temperature, and concentration of the premixed gases at the inlet are the same for both catalytic and non-catalytic cases. Similarly, the outflow is given for both catalytic and non-catalytic cases. This is because the combustion reaction occurred in the pore and on the surface of the solid skeleton of the catalytic porous burner, and more heat was released on the surface of the catalytic porous skeleton. Whereas, the combustion reaction only occurred in the pore of the inert porous, and all heat was released by gas phase (homogeneous) reactions for the inert porous burner, which led to higher gas temperature.

### Influence of Burner Structure

**Figure 9:**
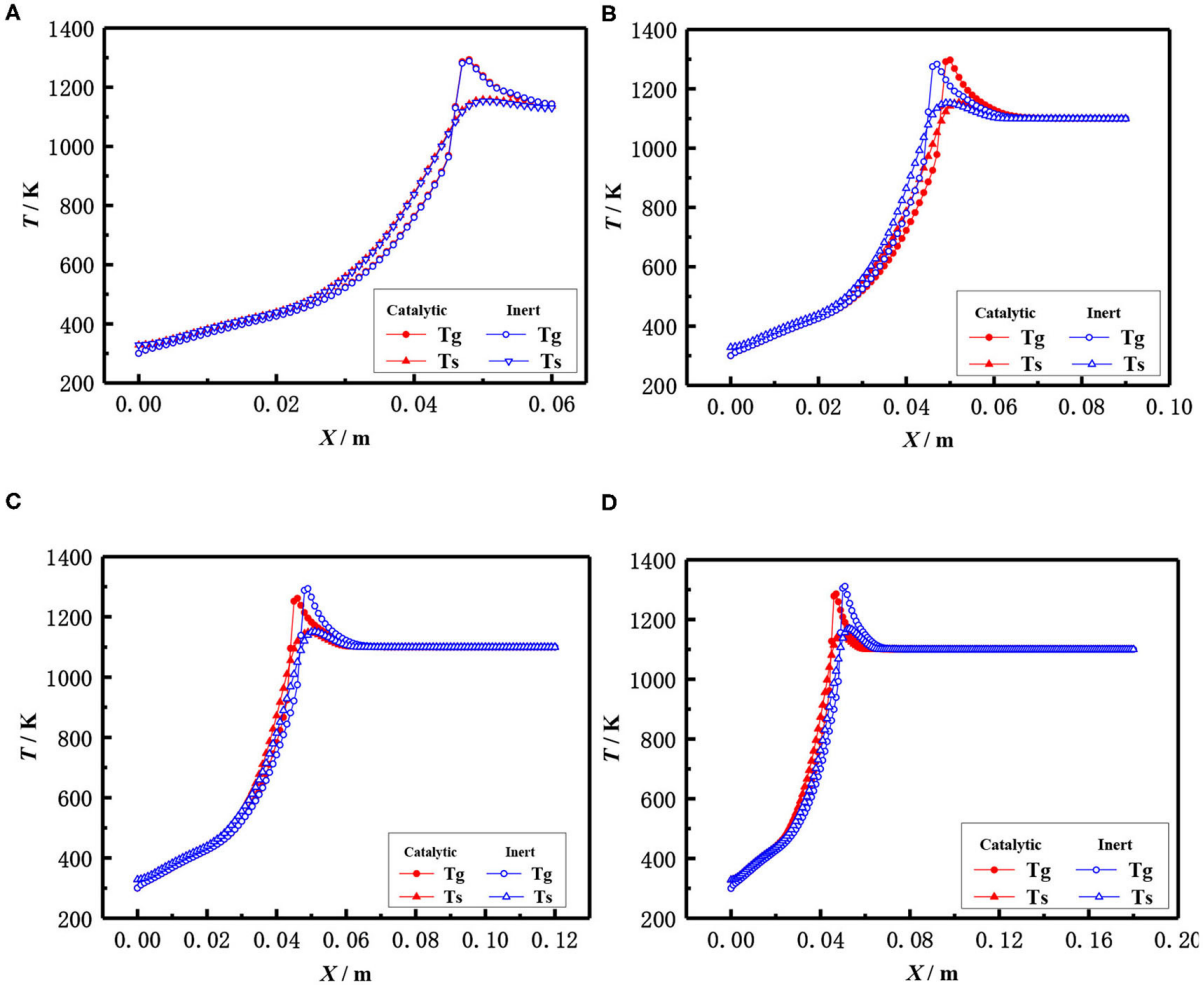
Effect of burner length on temperature distribution (φ = 0.30, S = 20 cm/ s). **(A)** 0.06 m, **(B)** 0.09 mm, **(C)** 0.12 m, and **(D)** 0.18 m.

The influence of the length of porous media on the flame temperature distribution for both catalytic and the inert porous burner at the equivalence ratio of φ = 0.30 and inlet velocity of S = 20 cm/s is shown in Figure 9, which indicates that the distance from the flame location to the burner inlet is almost constant with the increasing length of the porous media for both the catalytic and inert porous burner, while the relative position of the flame location moved toward the upstream. The flame was located at 5 cm from the inlet for different lengths of the burner. The length of porous media had a minor impact on the flame location of the burner, while the relative flame location was moved toward the upstream. The pressure drop was increased with the increase of solid skeleton length.

The flame locations and flame temperature distributions almost coincide with each other for the catalytic and inert porous burners when the length of the porous skeleton is shorter (60 mm). The distance from the flame location to the burner inlet was slightly increased, and the flame location of the inert porous burner slightly moved to the downstream, with the increasing length of the porous media for the inert porous burner. On the contrary, the flame location of the inert porous burner first moved toward the upstream and then toward the downstream when the length of the porous media was increased for the catalytic porous burner. The combustion reaction occurred in the pore and on the surface of the solid skeleton for the catalytic porous burner and more heat was released on the surface of the catalytic porous skeleton. However, more heat was released for the catalytic porous burner and relatively less heat was transferred from the combustion zone to the upstream by conduction and radiation through the solid skeleton when the flame was located at the center of the combustion chamber.

**Figure 10:**
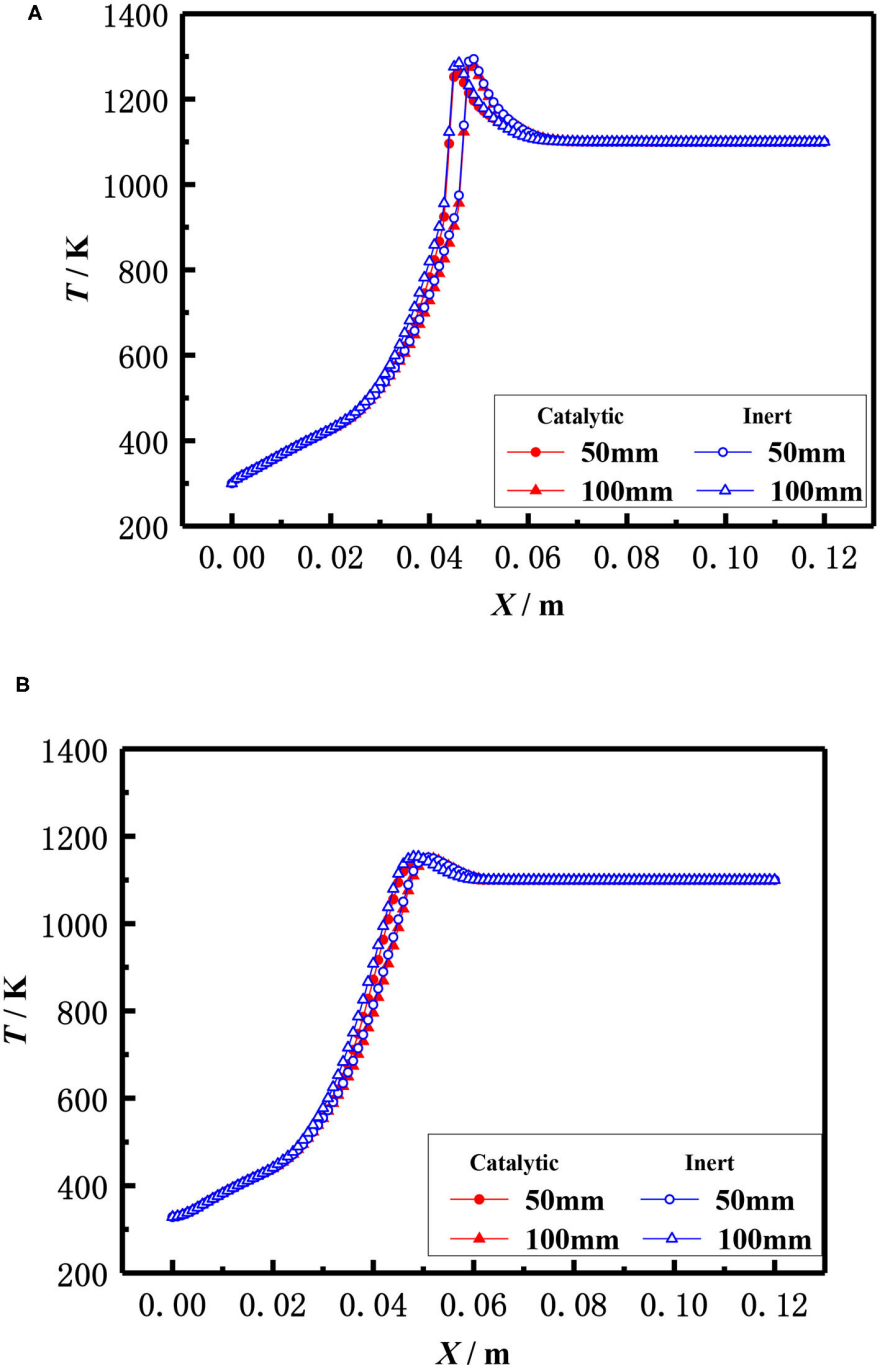
Effect of burner diameter on temperature distribution (φ = 0.30, S = 20 cm/s). **(A)** Gas temperature. **(B)** Solid temperature.

The influence of the burner diameter on the flame temperature distribution for both the catalytic and inert porous at the equivalence ratio of φ = 0.30 and inlet velocity of S = 20 cm/s is shown in Figure 10. The flame temperature distributions of the inert porous burner were similar and the flame slightly moved to the upstream for the inert porous burner, while the flame of the catalytic porous burner slightly moved to the downstream with an increase in burner diameter.

### Influence of Operating Parameters

**Figure 11:**
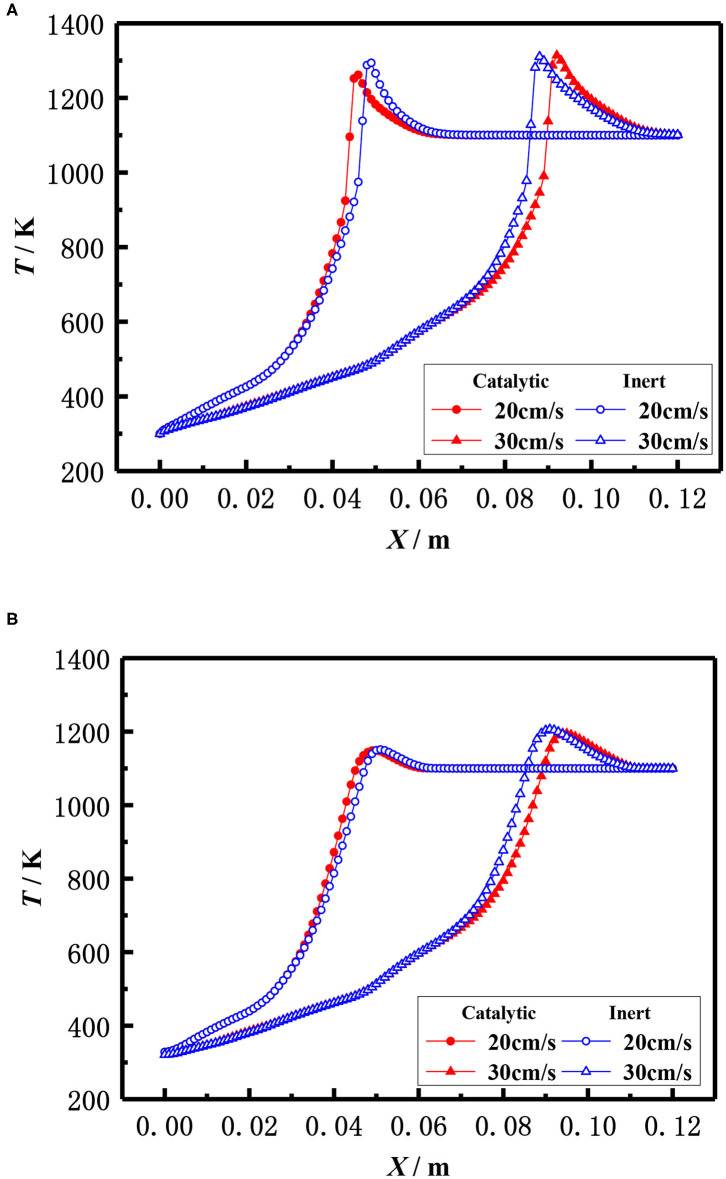
Effect of flow velocity on temperature distribution (φ = 0.30). **(A)** Gas temperature. **(B)** Solid temperature.

The influence of inlet velocity on the flame temperature distribution in catalytic and inert porous media burner at the equivalence ratio of φ = 0.30 is shown in Figure 11. The flame moved toward the exit and the flame temperature slightly raised with the increase of inlet velocity for both the catalytic and inert porous burner. More heat was lost to the environment by exhaust gases due to the higher temperature when the flame moved to the burner exit, resulting in a larger temperature gradient from the reaction zone to the exit. The flame location of the catalytic porous burner was far away from the exit than that of the inert porous burner at the lower inlet velocity (20 cm/s), whereas the flame location of the catalytic porous burner was much closer to the burner exit at a higher velocity (30 cm/s). The flame position of the catalytic porous burner was more sensitive to the flame velocity compared to the inert porous burner. This is because the combustion reaction occurred in the pore and on the surface of the solid skeleton for the catalytic porous burner; whereas the combustion reaction only occurred in the pore of the inert porous and all the heat was released by gas phase (homogeneous) reactions for the inert porous burner. The combustion reaction on the surface of the catalytic solid skeleton is more sensitive to the flame velocity due to full contact.

**Figure 12:**
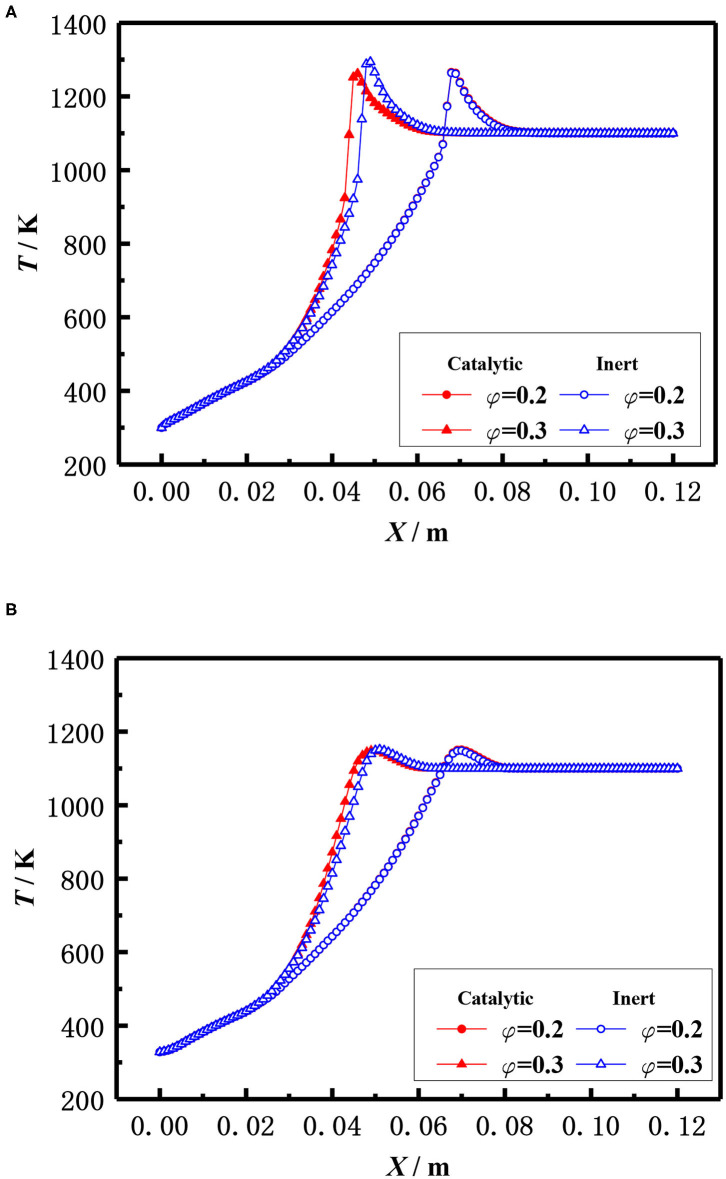
Effect of equivalence ratio on temperature distribution (S = 20 cm/s). **(A)** Gas temperature. **(B)** Solid temperature.

The influence of the equivalence ratio on the flame temperature distribution in catalytic and inert porous media burner at the inlet velocity of 20 cm/s is shown in Figure 12. The flame moved toward the upstream and the flame temperature slightly raised with the increase of equivalence ratio for both the catalytic and inert porous burner. The flame temperature distribution was almost identical at lower equivalence ratio (φ = 0.20), whereas the flame location of catalytic porous burner was much closer to the burner inlet at the equivalence ratio (φ = 0.30).

### Influence of Porous Media Parameters

**Figure 13:**
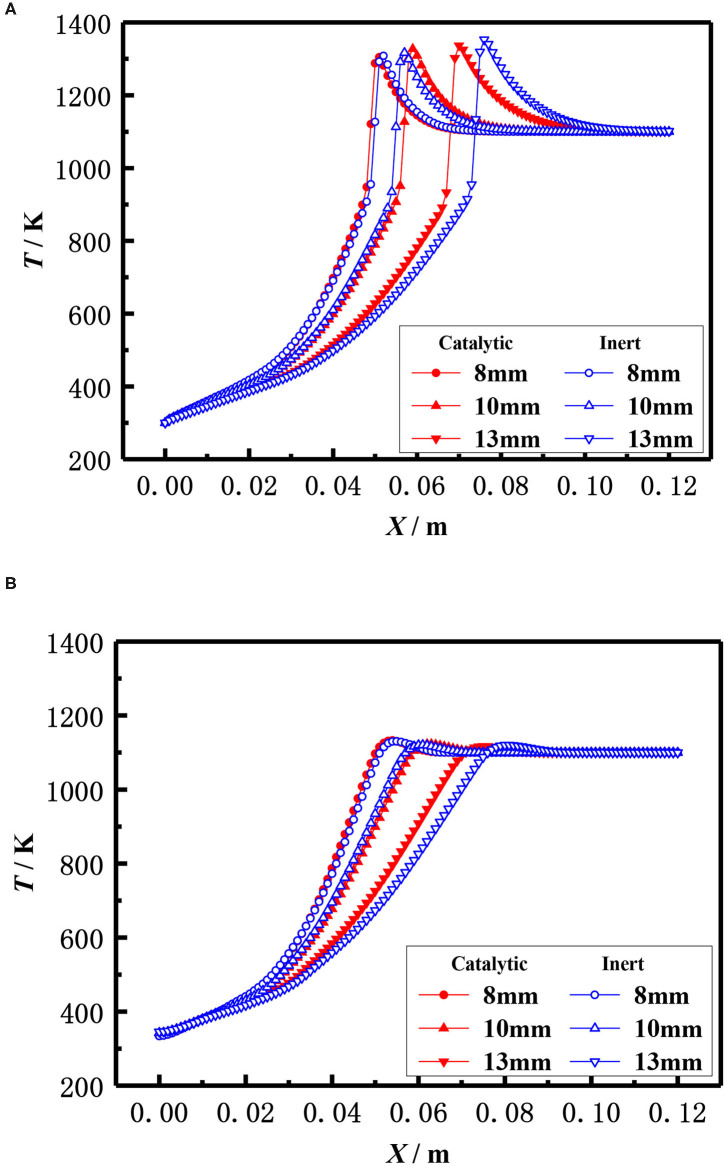
Effect of ball diameter on temperature distribution (φ = 0.30, S=20 cm/s). **(A)** Gas temperature. **(B)** Solid temperature.

The effect of the pellet diameter on the flame temperature distribution for catalytic and inert porous burner with equivalence ratio φ = 0.30 and inlet velocity S = 20 cm /s was shown in Figure 13, which illustrates that the flame moved toward the burner exit with the increasing diameter of the packed pellets at lower equivalence ratio. This is because the flame location relied on the heat that transferred from the reaction zone to the upstream by radiation of solid pellets and carried by the reaction gas to the downstream. The flame moved toward the upstream with the increase of packed pellet diameter. This was due to the heat transferred from the reaction zone to the upstream is significant as the lower radiative extinction coefficient and higher flame temperature at the larger equivalence ratio. The flame moved toward downstream with the increase of packed pellet diameter due to the larger heat carried from the reaction zone to the downstream by reactive gas, as there was a lower flame temperature at the smaller equivalence ratio. Furthermore, the flame location of the catalytic porous burner was far away from the burner outlet than that of the inert porous burner for the same diameter of packed pellets. This is because the chemical reaction on the surface of porous catalyst results in the increasing temperature of packed pellets and the higher heat transferred from the reaction zone to the upstream. It is indicated that the flame stability limits of the catalytic porous burner are wider than that of the inert porous burner.

**Figure 14:**
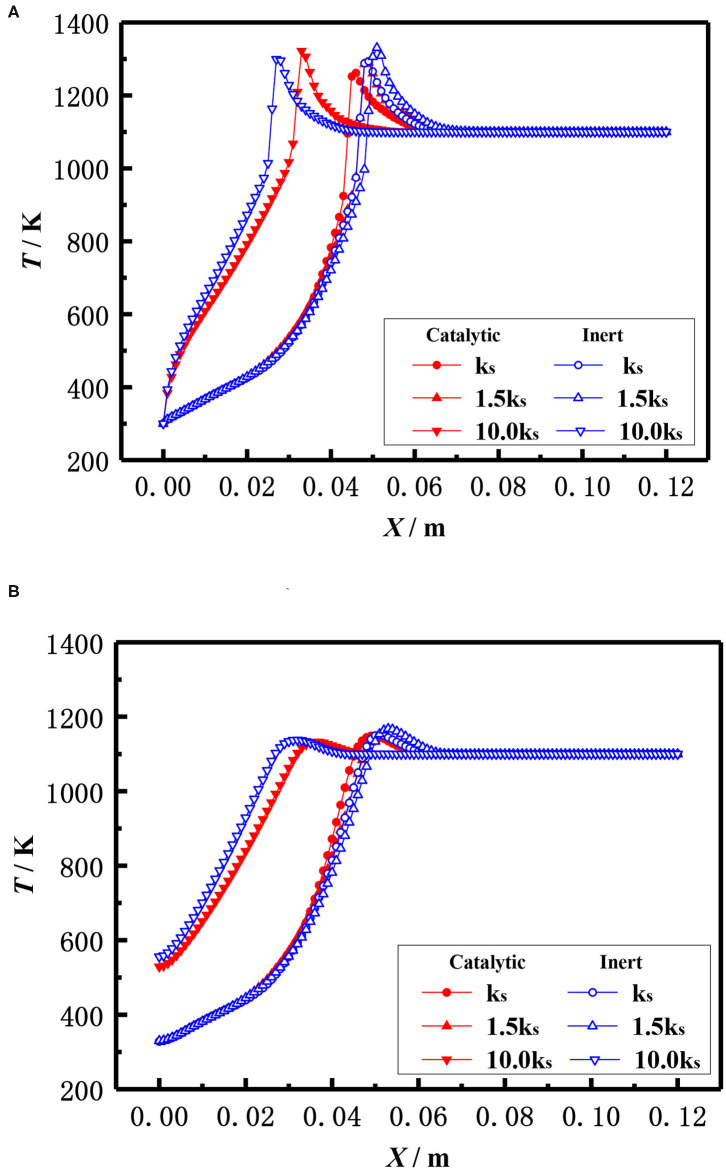
Effect of thermal conductivity on temperature distribution (φ = 0.30, S = 20 cm/s). **(A)** Gas temperature. **(B)** Solid temperature.

In addition to the diameter of packed pellets, the material, namely the thermal conductivity of packed pellets also affects the flame location of the burner. The effect of the thermal conductivity of the pellets on the flame temperature distribution of the catalytic and inert porous media burners with the equivalent ratio φ = 0.3 and the inlet velocity S = 20 cm/s is shown in Figure 14. The results showed that the flame location of both catalytic and inert porous burners moved from downstream to upstream with the increased thermal conductivity of the pellets. Compared with the inert porous burner, the flame location of the catalytic porous burner was mildly changed with the increase of thermal conductivity.

## Conclusions

In this paper, the premixed combustion of methane/air mixture in catalytic porous media and inert porous media was numerically investigated. The main conclusions are as follows:

1. The flame location of the catalytic porous burner was closer to the burner inlet than that of the inert porous burner at the lower inlet velocity (20 cm/s), whereas the flame location of the catalytic porous burner was much closer to the burner exit at the higher velocity (30 cm/s). The difference of flame location between catalytic and inert porous burner becomes smaller with the increase of inlet velocity. The flame location of the catalytic porous burner was more sensitive to the flame velocity compared to the inert porous burner. The flame temperature distribution was almost identical at lower equivalence ratio (φ = 0.20), whereas the flame location of the catalytic porous burner was much closer to the burner inlet at the equivalence ratio (φ = 0.30). The difference of flame temperature distribution between the catalytic and inert porous burner becomes larger with the increase of equivalence ratio.
2. The flame moved toward the burner exit with the increasing diameter of the packed pellets at a lower equivalence ratio. The flame location of both catalytic and inert porous burner moved from downstream to upstream with the increased thermal conductivity of packed pellets. Compared with inert porous combustion, the flame location of catalytic porous combustion is insensitive to thermal conductivity.
3. The distance of the flame location to the burner inlet is almost constant with the increasing length of the porous media for both the catalytic and inert porous burner, while the relative position of the flame location moved toward the upstream. The flame slightly moved to the upstream for the inert porous burner, while the flame of the catalytic porous burner slightly moved to the downstream with the increasing burner diameter.

In the present paper, the specific surface area of the catalytic porous media was calculated by the packed bed of inert pellets due to a lack of relevant formula. However, the specific surface area of the catalytic porous was much larger than that of inert pellets due to the complex catalyst loading method and procedure. The effect of the specific surface area of the catalytic porous on the performance of methane/air combustion will be investigated in future studies.

## Data Availability Statement

The raw data supporting the conclusions of this article will be made available by the authors, without undue reservation, to any qualified researcher.

## Author Contributions

HG and XF simulated the two dimensional model and wrote the manuscript. SZ analyzed numerical results and processed the data. CZ built the physical model and governing equations. All authors contributed to the article and approved the submitted version.

## Conflict of Interest

The authors declare that the research was conducted in the absence of any commercial or financial relationships that could be construed as a potential conflict of interest.
