# Supplementary material for: Two-Dimensional Numerical Study of Methane-Air Combustion Within Catalytic and Non-catalytic Porous Medium
Source: Front Chem. 2020 Nov 6;8:511792. doi: 10.3389/fchem.2020.511792 (PMC7677498; doi:10.3389/fchem.2020.511792)
Supplement: Supplementary file 1 [file Data_Sheet_1.docx]

**NOMENCLATURE**

| $\text{A}$ | pre-exponential factor |
| --- | --- |
| $a_{cat}$ | Catalytic porous media surface area, $m^{2}$ |
| $c_{s}$ | Specific heat of the pellet, $J/(kg\cdot K )$ |
| $c_{g}$ | specific heat of the mixture at constant pressure, $J/(kg\cdot K )$ |
| $C$ | concentration |
| $d_{p}$ | diameter, $m$ |
| $D$ | diffusion coefficient, $m{}^{2}/s$ |
| $E_{a}$ | activation energy, $kJ/mol$ |
| $E_{s}$ | activation energy of catalytic reaction, $kJ/mol$ |
| **$h_{v}$** | volumetric heat transfer coefficient, $W/(m^{3}\cdot K)$ |
| $H_{k}$ | specific enthalpy of species$k$,$J/kg$ |
| $k$ | thermal conductivity , $W/(m\cdot K )$ |
| $k_{eff}$ | effective thermal conductivity, $W/(m\cdot K )$ |
| $\text{Nu}$ | Nusselt number |
| $p$ | Pressure, Pa |
| $\Pr$ | Prandtl number$\Pr=\frac{\rho_{g}c_{pg}\varepsilon ud_{p}}{\lambda_{g}}$ |
| $\mathrm{Re}_{p}$ | Reynolds number |
| r | radial coordinate, $m$ |
| $R_{r}$ | axial momentum equation |
| $R_{u}$ | universal gas constant, $J/(kmol\cdot K)$ |
| $R_{X}$ | radial momentum equation |
| $S$ | flow velocity,$cm/s$ |
| $T$ | temperature, $K$ |
| $u$ | velocity, $m/s$ |
| $V_{k}$ | diffusion velocity, $m/s$ |
| $W_{k}$ | molecular weight of gas, *g/mol* |
| $x$ | axial coordinate , $m$ |
| $Y_{k}$ | mass fraction |

**GREEK SYMBOLS**

| $\varepsilon$ | porosity |
| --- | --- |
| $\varepsilon_{r}$ | emissivity |
| $\mu$ | viscosity, $kg/(m\cdot s)$ |
| $\nu$ | kinematic Viscosity, $m^{2}/s$ |
| $\tau$ | time, s |
| $\rho$ | mixture density, $kg/m^{3}$ |
| $\sigma$ | Stefan-Boltzmann constant,  |
| $\varphi$ | equivalence ratio |
| $\omega^{h}$ | molar consumption rate per unit volume for gas-phase reaction, $kmol/(m^{3}\cdot s)$ |
| $\omega^{S}$ | molar consumption of the catalyst for surface reaction, $kmol/(m^{2}\cdot s)$ |
|  | the molar rate of the k th component*,* $kmol/(m^{3}\cdot s)$ |

**SUBSCRIPTS**

| CH_4_ | methane |
| --- | --- |
| eff | effective |
| fu | fuel |
| g | gas |
| $in$ | inlet, initial |
| $k$ | species |
| loss | heat loss |
| $O_{2}$ | oxygen |
| ox | oxygen |
| rad | radiation |
| s | solid |
| Sur | surroundings |
| Wall | wall surface |
|  |  |
